# RAPT: A Robust Attack Path Tracing Algorithm to Mitigate SYN-Flood DDoS Cyberattacks

**DOI:** 10.3390/s23010102

**Published:** 2022-12-22

**Authors:** Zakwan AlArnaout, Nour Mostafa, Samer Alabed, Wael Hosny Fouad Aly, Ahmed Shdefat

**Affiliations:** 1 College of Engineering and Technology, American University of the Middle East, Egaila 54200, Kuwait; 2 Biomedical Engineering Department, School of Applied Medical Sciences, German Jordanian University, Amman 11180, Jordan

**Keywords:** DDoS mitigation, SYN-flood attack, IP traceback, cybersecurity

## Abstract

In the recent past, Distributed Denial of Service (DDoS) attacks have become more abundant and present one of the most serious security threats. In a DDoS attack, the attacker controls a *botnet* of *daemons* residing in vulnerable hosts that send a significant amount of traffic to flood the victim or the network infrastructure. In this paper, a common type of DDoS attacks known as “TCP SYN-Flood” is studied. This type of attack uses spoofed Internet Protocol (IP) addresses for SYN packets by exploiting the weakness in Transmission Control Protocol (TCP) 3-Way handshake used by the TCP/IP suite of protocols, which make the web servers unreachable for legitimate users or even worse, it might lead to server crash. In this paper, a resilient, efficient, lightweight, and robust IP traceback algorithm is proposed using an IP tracing packet for each attack path. The proposed algorithm suggests that edge routers—where the attack starts from—observe the traffic pattern passing through, and if the observed traffic carries the signature of TCP SYN-Flood DDoS attack and a high percentage of it is destined to a particular web server(s), it starts the tracing process by generating an IP trace packet, which accompanies the attack path recording the routers’ IP addresses on the path between the attacker/daemon and the victim, which can extract the path and react properly upon receiving it by discarding any SYN packets originating from that attacker/daemon. To our knowledge, this is the first research that efficiently traces these kinds of attacks while they are running. The proposed solution has low computation and message overhead, efficient detection and tracing time, and converges in near optimal time. The results are validated using extensive simulation runs.

## 1. Introduction

In the early 2000s, communication networks faced many challenges in providing reliable levels of security by achieving the well-known three pillars of information security, i.e., confidentiality, integrity and availability [1]. Among these, availability has been one of the largest challenges. Denial of Service (DoS) attacks have been a major challenge in the field of cybersecurity for more than 20 years. These attacks drain the network and server(s) resources of a targeted system; as a result, this prevents legitimate users from accessing the network/server resources. DDoS attacks operate on a larger scale in which a large number of vulnerable and/or compromised hosts making up a botnet [2] are exploited to initiate parallel attacks simultaneously. DoS/DDoS attacks on the Internet have increased in frequency, rigor and complexity, where they have become one of the most dangerous threats over the Internet. The easiness and cheap methods of performing these attacks have allowed them to exponentially grow, reaching magnitudes over 1.7 Tbps in 2018 [3]. Therefore, it is a paramount objective to understand the dynamics of these kinds of attacks to develop real-time and efficient mitigation algorithms. In traditional networks, DDoS attacks can be categorized into volumetric, protocol exploitation and application attacks [4]. Attackers have developed tools to coordinate distributed attacks from several separate locations. Unfortunately, the mechanisms for dealing with DoS/DDoS attacks are perhaps one of the least researched topics in network security. There are many reasons why there are no fundamental solutions to DoS/DDoS attacks due to the nature of the TCP/IP protocol suite that is not resilient to such attacks because, when TCP/IP was designed, security threats were not taken into consideration. Confidentiality and integrity were well-researched, and strong encryption protocols and algorithms have been found to provide robust defense against attacks on confidentiality and integrity. On the other hand, availability has received less research because security threats to availability began to emerge in the mid-1990s and its danger, complexity and negative effects intensified in the early 2000s attacks, which led to the disruption of huge servers and websites [5]. The contribution in this paper is a two-fold algorithm for tracing SYN-Flood attacks: the first is deployed on edge routers at the daemons’ side, while the second is deployed on the core routers. The algorithm is characterized to be resilient, lightweight, and robust using IP traceback packets. The algorithm detects the potential of an attack based on recorded statistical information at the edge routers. When the SYN traffic surges towards a particular destination, the edge router compares the traffic surge with historical access patterns, and a decision will be taken to initiate the IP traceback packet that accompanies the generated attack traffic. This IP traceback packet will be used to record the IP addresses of the routers between the source and the victim. The efficiency and feasibility of the algorithm are validated using extensive simulation experiments and compare the performance of the proposed algorithm with the related algorithm. The obtained results show that the proposed algorithm is robust, effective, efficient, and reacts rapidly against DDoS attacks.

### 1.1. Motivation

Upon the design of the Internet Protocol (IP) by the Advanced Research Projects Agency Network (ARPANET) in the 1970s, the focus was majorly on achieving seamless communication between different entities. Therefore, at that time, security was not an objective by its own in the protocol design. This eventually has made the communication vulnerable to attacks that exploit loopholes in the TCP/IP protocol stack. Nowadays, Internet has become an indispensable communication and service provision platform in our daily life. Unfortunately, we are witnessing a dramatic increase of attack incidents where every host machine connected to the Internet is liable of becoming attacked. It is not only the Internet users that are annoyed and prone to cybersecurity attacks causing failures to the Internet service access, but the commercial/governmental entities are also suffering from financial losses, privacy issues and bad publicity. DDoS attacks, in particular, have caused severe disruptions to the Internet services in recent years [6]. These kinds of attacks are considered among the hardest security threats to address because they are easy to deploy and implement; moreover, they are hard to prevent and trace [7]. Since multi-source DDoS attacks are much more complicated than a single-source DoS attack, DDoS attacks are considered as the target problem in this work. Recent incidents have demonstrated so many vulnerabilities and breaches to the IP exploiting DDoS attacks [8]. Therefore, there is a pressing need to conduct research in the field of cybersecurity to devise an efficient and robust scheme to defend against DDoS attacks.

### 1.2. Research Methodology

**Figure 1:**
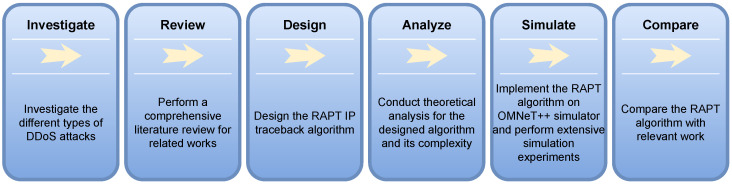
Research methodology phases.

In this paper, the research methodology is divided into phases as illustrated in Figure 1. These phases are discussed as follows:

1. Different types of DDoS attacks are investigated and explored. The investigation is narrowed down to focus on SYN-Flood DDoS attacks;
2. A comprehensive literature review has been conducted to find the strength and limitations of previous works;
3. A careful design for the IP traceback algorithm has been proposed considering scenarios for DoS and DDoS attacks. The design periodically collects statistical information about traffic forwarded at the edge routers where the algorithm decides upon initiating the IP traceback or not. Two unique features identify the scheme. The first is the reliability of path reconstruction using hop-by-hop TCP connections. The second feature is the use of one trace packet per attack path, which makes it lightweight by avoiding the injection of extra traffic overhead in the network;
4. Theoretical analysis was performed to find the complexity of the proposed algorithm and analyzing the convergence time of it;
5. Extensive simulation runs were conducted to validate the correctness of the algorithm. Each data point represents the average of 15 simulation runs;
6. The proposed algorithm is compared with a relevant one using different performance metrics.

     The rest of the paper is organized as follows: Section 2 reviews the related work. The proposed attack path tracing algorithm is presented in Section 3. Section 4 discusses the theoretical analysis, simulation methodology, and performance evaluation. Finally, Section 5 concludes the paper, and future work is provided.

## 2. Related Work

In this section, a review of the main contributions related to IP traceback algorithms is provided. Tracing the DDoS source is one of the effective methods to prevent DDoS attacks. The existing detection models against DDoS are primarily following a two-phase procedure that performs detection and classification to determine the malicious sources and then mitigating the attack by performing access control. These models deal with the two phases as two separate units, making it hard to generate mitigation strategies in reference to the conditions of special attacks. Below, a detailed discussion of the related work in each phase is provided.

### 2.1. Detection and Classification Approaches

The detection and classification approaches can be subdivided into the below categories:

1. **Statistical Approaches**: Statistical models are built on benign and malicious DDoS traffic, which are then applied to detect and classify DDoS attacks. In [9], DDoS Shield classifies DDoS attacks based on the exploited application workload parameters. The threshold values are preset on the workload parameters based on the measurements and labels the behaviors exceeding the thresholds as malicious. Ref. [10] proposed a scheme that detects floods of HTTP GET by modeling the webpages’ browsing order and finding the correlation between browsing time and the pages’ information size. In general, statistical approaches are efficient and controllable providing good accuracy in discovering simple DDoS attacks such as HTTP flood and slow DDoS attacks; however, they might not be efficient in handling complicated attacks.
2. **Learning Approaches**: With the machine learning algorithms’ advancement and ability to provide intelligent solutions, many researchers are harnessing these techniques on big data for detecting DDoS attacks. In [11], the authors proposed a three-layer feed-forward neural network that detects DDoS attacks using extracted features from the packets’ header fields. Ref. [12] applied Stacked AutoEncoder, which is a deep learning scheme that aims to receive high-level features and generates features from web server logs building a logistic regression classifier to determine DDoS attacks. ARTP [13] detects DDoS attacks by leveraging the K-means algorithm and conducting analysis on features such as request’s interval, chain context, and length. To summarize, leveraging machine learning techniques to identify DDoS have mixed results depending on the feature extraction approach, the scheme’s design, and the learning algorithm.
3. **Markovian Approaches**: A Markov model is defined as a stochastic model that models randomly changing events in probability theory [14]. It works by assuming that the future state depends on the current state only, and the next state can be inferred by conducting probability analysis on its past. The works in [15,16,17,18] track the correlated behavior of users and utilizes hidden semi-Markov model with a random-walk graph for tracing the DDoS attacks. Markov-based approaches are the state of the art because DDoS attacks are stateful. To address stateful issues, Markov-based approaches can provide fine-grained and highly accurate detection and classification results.
4. **Probabilistic Packet Marking Approaches**: In [19,20], the scheme suggests that routers randomly mark IP packets using an edge identifier, which is the start and end points of a single hop in the packet path. If enough packets are sent, the victim can construct the attack path through the parameterized packets. In this scheme, it is suggested that the information to be encoded in the identification field of the IP header. However, the modification of the identification field in the IP header could lead to the failure in assembling the fragments completely. In [21], Traffic-Aware Probabilistic Packet Marking (TAPPM) was proposed, which is a collaborative DDoS mitigation framework based on traffic-aware probabilistic packet marking. It enables the victim to select some of the hit routers as collaborators to mitigate attack traffic depending on the traffic distribution. In [22], the authors proposed a solution to the Interest Flooding Attack (IFA) in Named Data Networking. The solution leverages hypothesis testing theory to develop a Generalized Likelihood Ratio Test adapted to evolved IFA attacks. The work presented in [23] suggests an overview of the main cloud vulnerabilities, along with solutions proposed for the H2020 CLARUS project. It provides monitoring techniques for intrusion detection and prevention, including attack tolerance mechanisms. In [24], a trusted multidomain DDoS detection method based on federated learning was proposed. It works by dividing the types of DDoS attacks into sub-attacks where, in each domain, a design of the federated learning dataset for DDoS detection is made and used to realize a comprehensive detection method. Packet marking schemes were proposed in works such as the spatial marking technique [25], deterministic packet marking [26], and marking-on-demand scheme [27]. These schemes insert marks into a predefined space of the IP header of the packets. However, the uncertainty of IP marking leads to information loss at a certain tracing failure probability. Moreover, these marking methods cause an increase in overhead when an attack occurs. In [28], the authors used blockchain to support data accountability and tracking sources. The study focuses on the production line of IoT devices but does not use the characteristics of blockchain to track DDoS attacks.

### 2.2. Mitigation Approaches

There are mainly two directions in the research of DDoS mitigation approaches [5]. The first aims to mitigate attacks on the victim’s side, one of the methods is by blocking automated application requests utilizing user puzzles such as the works proposed in [29,30], where IP tables are setup for Intrusion Detection Systems (IDS) rules [31]. Although message-level mitigation granularity can be achieved, the mitigation may be less efficient as the malicious packet must be received by the victim before mitigation, and the system is still vulnerable to DDoS attacks. The second direction is by mitigating DDoS attacks in the network. Once the victim has identified the source of the attack, traffic filtering or rerouting schemes such as the works in [5,32,33] that can be used to mitigate the attack within the network without consuming resources on the victim’s part. However, traffic from benign IPs can also be filtered, which can cause significant collateral damage. Our approach includes both victim-side and in-network mitigation for efficient and effective defense against DDoS attacks.

To summarize, since the introduction of DDoS attacks, tremendous efforts have been put into trying to find efficient solutions to the problem; however, no specific solution was designed specifically for SYN-Flood attacks. To our knowledge, no optimal solution has emerged so far. This section has presented a summary for a number of proposed solutions that have some but not all of the required characteristics. The aforementioned works have drawbacks in terms of the administrative burden, the extra injected network traffic, backward compatibility with the TCP/IP protocol suite, and the overhead on the victim itself.

## 3. The Proposed Robust Attack Path Tracing Algorithm

In this section, the design of the proposed Robust Attack Path Tracing (RAPT) algorithm is discussed. Firstly, the kind of attacks the algorithm deals with is identified; secondly, a discussion of the needed features and assumptions on which the algorithm is based on is provided, and lastly the algorithm design is introduced.

### 3.1. Attack Type

The DDoS attack that the proposed algorithm aims to stop and handle is of the flooding category. The reason behind this is coming from the fact that a significant portion of DDoS attacks falls under this category [34]. In 2019, it was found that 36% of DDoS attacks are UDP Flooding and 14% SYN Flooding attacks in the year 2019 alone [35]. Ideally, the tracing scheme should be able to identify the source of any piece of information sent over the network. Within the IP protocol framework, a packet is the smallest atomic unit in the data and any smaller division of the data (e.g., byte) is contained in an IP packet. A typical tracing system must accurately identify the source of any random packet, and any larger data unit or data stream that can be separated by searching for any particular packet containing data within that stream.

### 3.2. Assumptions and Features

**Figure 2:**
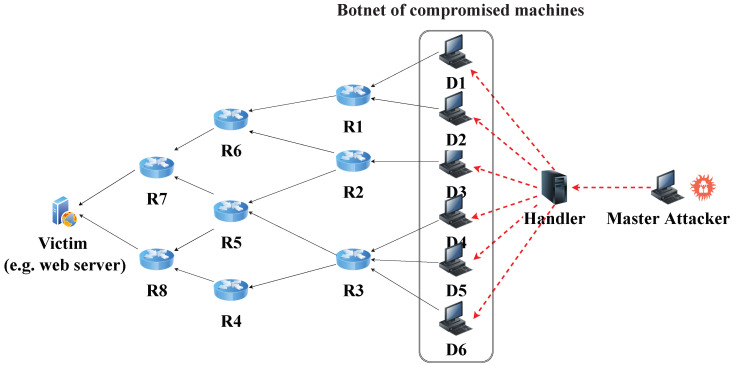
Potential DDoS attacks from six different daemons targeting the same victim.

The algorithm assumes that any router within the attack path is willing to cooperate and be enabled with the RAPT algorithm; to this end, it becomes clear that the algorithm does not only have to distinguish the source of the attacking packet, but also to identify the entire path between the source and the victim. If a path can be traced through any number of flawless or faulty routers, it must either terminate at the source or pass through a faulty router. This is why it is important to construct the attack path that contains every router in which the potential daemon packet passes through its journey from source to victim. Additionally, since multiple packets may be injected into the network from different sources (daemons), the tracing algorithm must generate an attack graph composed of the attack path for each instance of the attack packets delivered to the victim. Figure 2 describes the network as seen by the victim with a possible attack on the victim from daemons D1 through D6 forming five different attack paths represented by the tuples {R1, R6, R7}, {R2, R6, R7}, {R2, R5, R7}, {R2, R5, R8}, and {R3, R4, R8}. The daemons are assumed to collude together to initiate a synchronized attack targeting the victim.

Although there are several solutions proposed for DDoS attacks phenomenon in the literature, the features of the proposed solution make it unique. The objective of the RAPT algorithm is to find a feasible solution through an IP traceback approach to detect the source of the attack. The following criteria must be met in order to have a feasible solution:

- It must track down and stop the SYN-Flood attack;
- It must be robust against distributed attacks;
- The detection time must be near-optimal;
- It should not dramatically affect the victim and/or network performance; and
- Administrative cost should be minimal.

### 3.3. RAPT Algorithm Design

The RAPT algorithm is based on periodic logging of statistical information about the SYN packets relayed through the edge routers and comparing the frequency of SYN packets for the current period with the previous period. This information is recorded in certain tables as shown in Table 1 below. The edge router uses this information to deduce and make an informed decision whether to initiate the tracing algorithm or not. A record in the log table will include the following information:

1. Destination IP address of the packet;
2. Frequency ($f_{t}$) and ($f_{t-1}$) of received SYN packets during the periods *t* and t−1, respectively, noting that $f_{t}$ will become $f_{t-1}$ for the next period;
3. Relative change in Frequency ($\Delta f_{t}$) of SYN packets;
4. Cumulative mean of Frequency ($\mu f_{t}$) in SYN packets.

**Table 1:** SYN packet logging table.

Destination IP Address	$f_{t-1}$	$f_{t}$	$\Delta f_{t}$	$\mu f_{t}$
142.250.185.132	7	20	1.86	84
20.50.169.216	126	432	2.43	170
87.236.232.169	531	274	−0.48	243
195.26.133.182	376	248	−0.34	289

It should be noted that packet logging has been used in works such as [36,37]. Moreover, the observed destination IP addresses are for popular web servers that are prone to attacks for their availability importance. The RAPT algorithm does not consider end hosts or unpopular web servers. When the edge router forwards a SYN packet (or more) towards a destination IP address for the first time, the router will build a new entry for that address and forwards the packet along the routing path to the next router. The logging table does not keep its entries indefinitely, and it follows the Least Frequently Used (LFU) caching policy during each period for evicting entries when it becomes full. LFU was selected because the least used entry means the destination IP address is least targeted. The RAPT algorithm is divided into two parts, the first part is implemented on the edge routers, while the second part is implemented on the core routers.

#### 3.3.1. At the Edge Router $R_{i}$

In this subsection, a detailed discussion is provided on how the RAPT algorithm (see Algorithm 1) works on the edge router. Table 2 provides the notation used throughout the algorithm. In the RAPT algorithm, the edge routers periodically monitor two parameters, namely, (i) The relative change in frequency ($\Delta f_{t}$); and (ii) the cumulative mean of frequency ($\mu f_{t}$). Then, the edge router $R_{i}$ performs the following steps:

1. At the end of the current *t* period, the edge router compares $\Delta f_{t}$ and $\mu f_{t}$ (see Equations (1) and (2) below) against two thresholds α and β, respectively. If both are exceeding these two thresholds, this might indicate that the SYN traffic is malicious and a potential attack might be active. Therefore, the router must create a trace packet called *TracePack*, which will pass through all the routers between the victim and the attacker/daemon including edge and core routers. The enroute routers record their IP addresses on the payload of TracePack, which is used by the victim to extract the entire attack path:
  (1) $$\begin{array}{c} \Delta f_{t}=\frac{f_{t}-f_{t-1}}{f_{t-1}} \end{array}$$
  (2) $$\begin{array}{c} \mu f_{t}=\frac{f_{t-1}+n.\mu f_{t-1}}{n+1} \end{array}$$
2. Pick one of the SYN packets randomly named *RandPack* from the incoming SYN traffic destined to the potential victim;
  (a) If the packet is a fragment, its identifier will be the value stored in the identification field of the IP header;
  (b) Otherwise, the unused identification field (set to 0) will be used such that the router generates a pseudo random number as a function of time to be used as an identifier for the packet and write it to the identification field;
3. $R_{i}$ generates a trace packet called TracePack with the following information:
  (a) Source address being $R_{i}$s IP address;
  (b) Destination address is RandPack’s destination address (victim’s IP address);
  (c) Append to the payload of TracePack the source address of RandPack, identifier of RandPack, and $R_{i}$s IP address. The reason behind appending RandPack’s ID field is because the next-hop router needs to recognize RandPack from other flowing packets. This is ensured by forwarding TracePack ahead of RandPack, which allows $R_{j}$ to capture RandPack;
4. $R_{i}$ sends TracePack to the next-hop $R_{j}$ using hop-by-hop TCP connections [38] to guarantee the delivery of TracePack;
5. Upon receiving the acknowledgement of TracePack’s arrival to $R_{j}$, then $R_{i}$ sends RandPack to the next-hop $R_{j}$ similarly using hop-by-hop TCP connections.

**Table 2:** Notation used in the RAPT algorithm.

Symbol	Meaning
Z	HotSet of IP addresses to be monitored against SYN-Flood attacks.
$f_{t}$	Frequency of received SYN packets during period *t*.
$f_{t-1}$	Frequency of received SYN packets during previous period t−1.
$\Delta f_{t}$	Relative change in Frequency of SYN packets.
$\mu f_{t}$	Cumulative mean of Frequency in SYN packets.
$R_{i}$	Current router (edge or core).
$R_{j}$	Next-hop router towards the victim.
*n*	Number of observed periods.
α	Threshold acceptable for $\Delta f_{t}$.
β	Threshold acceptable for $\mu f_{t}$.

**Algorithm 1:** Pseudo code for the RAPT algorithm at the edge router.
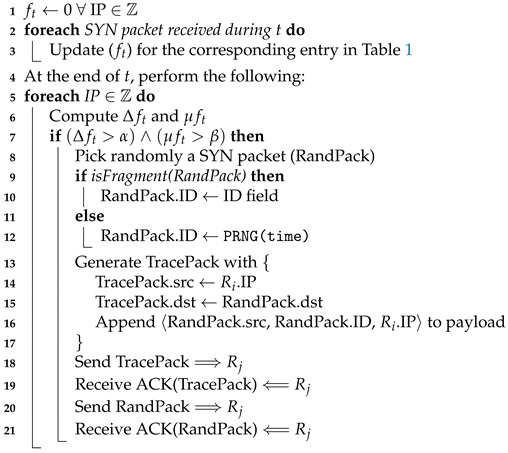


#### 3.3.2. At the Core Router $R_{j}$

In this subsection, a discussion is conducted on how core routers execute the RAPT algorithm related to them. The following elaborates on the steps in Algorithm 2:

1. When the core router $R_{j}$ receives a packet that requires an ACK, this indicates that the received packet could be a TracePack packet. Recall that lines 19 and 21 of Algorithm 1 demand a hop-by-hop TCP connections. To verify this, $R_{j}$ would lookup its Routing Information Base (RIB) for TracePack’s source IP address. If a match is found and it is one hop away, in addition, TracePack’s destination IP address is not any router’s IP address (i.e., the destination IP for TracePack is a host machine IP address). Given that these two conditions are satisfied, $R_{j}$ would infer that the received packet is a tracing packet:
2. $R_{j}$ replaces TracePack’s source IP address with its own IP address;
3. $R_{j}$ appends its IP address to the payload of TracePack;
4. $R_{j}$ sends an ACK to $R_{i}$ to acknowledge receiving TracePack;
5. The last four lines of Algorithm 2 are similar to the last four lines of Algorithm 1 that were already discussed.

**Algorithm 2:** Pseudo code for the RAPT algorithm at the core router.
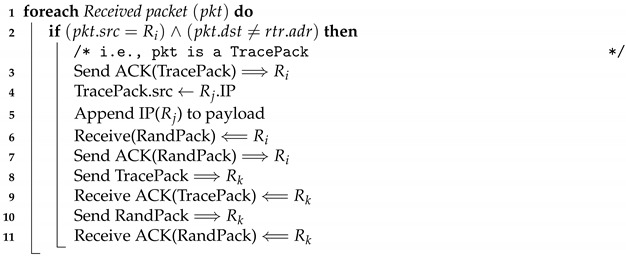


#### 3.3.3. At the Victim V

**Figure 3:**
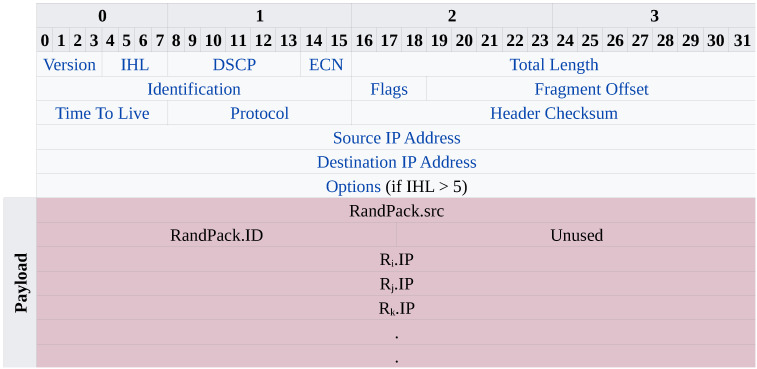
Structure of the payload in the TracePack packet received by the victim.

When TracePack reaches the victim, which is potentially under or after a DDoS attack, it can extract the attack path from the payload of TracePack, which contains the source IP address of the assumed attacker/daemon. It should be noted that the IP address might be spoofed and not necessarily leading to the daemon host itself; however, this helps the victim to decide on dropping connection requests from that IP address. Figure 3 depicts the structure of the payload in the TracePack packet. The extraction of the payload is performed as follows:

(i) 32 bits for the attacker’s source IP address;
(ii) 16 bits for the identification field of RandPack; and
(iii) The rest of the payload represents the IP addresses of routers in the path, each of which is 32 bits.

The victim can take an action of dropping SYN packets coming from this source or multiple sources in the case of the DDoS attack.

## 4. Performance Evaluation and Results Discussion

In this section, a theoretical analysis is provided; then, the proposed RAPT algorithm will be evaluated with patterns of traffic containing SYN-Flood DDoS attacks modeled and simulated, and tracing accuracy of the proposed algorithm is compared with a relevant one.

### 4.1. Theoretical Analysis

In this subsection, we compare theoretically the TAPPM [21] algorithm with the proposed RAPT algorithm. In order to prove how RAPT outperforms the TAPPM algorithm, we compare the aspects of convergence time and the path reconstruction cost between both of the algorithms.

#### 4.1.1. Convergence Time

The convergence time is the required time to reconstruct the paths and identify the attack sources completely. In this analysis, we consider the needed number of packets for path reconstruction as the convergence time.

1. **TAPPM**: Let ϕ be the marking probability for each of the routers, *d* be the distance between the router and victim, and Ψ be the probability, and we obtain the router’s information. The probability $\Psi_{TAPPM}$ of TAPPM is:
  (3) $$\begin{array}{c} \Psi_{TAPPM}=\phi {\left(1-\phi \right)}^{d-1} \end{array}$$
  Then, the convergence time $\Sigma {\left(m\right)}_{TAPPM}$ in terms of needed packet *m* for TAPPM will be:
  (4) $$\begin{array}{c} \Sigma {\left(m\right)}_{TAPPM}=\frac{ln(d)}{\phi {\left(1-\phi \right)}^{d-1}} \end{array}$$
2. **RAPT**: Due to the nature of RAPT, there will not be any omission, the probability to obtain the farthest routers’ information is thus dependent on ϕ. Therefore, the probability $\Psi_{RAPT}$ of RAPT is:
  (5) $$\begin{array}{c} \Psi_{RAPT}=\phi \end{array}$$
  The convergence time $\Sigma {\left(m\right)}_{RAPT}$ will be:
  (6) $$\begin{array}{c} \Sigma {\left(m\right)}_{RAPT}=\frac{ln(d)}{\phi } \end{array}$$
  Obviously, when 0 < ϕ < 1 and d > 1, then:
  (7) $$\begin{array}{c} \Psi_{TAPPM}\ll \Psi_{RAPT} \end{array}$$
  (8) $$\begin{array}{c} \Sigma_{TAPPM}\gg \Sigma_{RAPT} \end{array}$$

This gives evidence of how TAPPM needs many more packets than RAPT for convergence time.

#### 4.1.2. Path Reconstruction Cost

To validate the feasibility of RAPT algorithm, we evaluate the cost of path reconstruction and compare it with TAPPM. The complexity of an IP traceback algorithm is an important evaluation factor of it.

1. **TAPPM**: This algorithm needs to collect information fragments and perform hash functions on them upon path reconstruction; the algorithm complexity in DDos attack is $O(n^{2});$
2. **RAPT**: Given the pseudo code both in 1 and 2 at the edge routers and core routers respectively, we can analyze and find out that both of them are O(n) for both of Dos and DDoS attacks.

### 4.2. Scenario and Parameters’ Values

The simulator used for conducting the experiments is OMNeT++ simulator [39] with the INET Framework. The algorithm was coded and implemented on top of the INET Framework. The edge routers are running the OSPFv2 routing protocol, while the core routers are running the BGP routing protocol. At the application layer, two kinds of traffic are generated, Constant Bit Rate (CBR) acting as a background traffic and HTTP used for testing and evaluating the RAPT algorithm. Two sets of experiments were run on the implemented RAPT algorithm over the INET Framework. Each simulation scenario test was run for 15 times in a random distribution of traffic generation. The simulation results are averaged, and the results are based on a transient state system. The following metrics have been recorded:

1. *Detection time*: This is the time during which the edge router detects the possibility of an attack, and it is the period from the start of the simulation to the beginning of the tracing process;
2. *Number of SYN packets destined to the victim*: This represents the number of SYN packets destined to the victim from the beginning of the simulation until the start of the tracing process for each edge router;
3. *Tracing time*: The time it takes to trace the attack;
4. *Total processing time*: The total time elapsed by the routers on the attack path to carry out the tracing process;
5. *Convergence time*: The time taken by the algorithm to detect, trace and stop the attacks from all incoming paths until the victim receives legitimate SYN packets.

### 4.3. First Experiment Set with Different Traffic Loads

**Figure 4:**
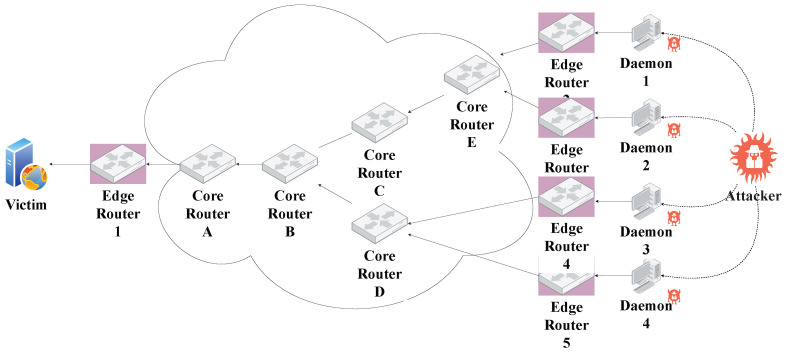
Partial network used in the simulation experiments.

In this experiment set, the simulator was run on a partial network as illustrated in Figure 4. Different loads of traffic (CBR and HTTP) generated at the edge routers 2 through 5, including both legitimate and malicious traffic, were considered. The data rate per link between routers was set to 100 Gbps. The simulator was run 15 times for four different traffic loads of 5000, 10,000, 20,000, and 40,000 packets generated from the edge routers. The number of observed periods *n* was set to 4, and the length of each period *t* was set to 20 s. The two thresholds α and β are set to 3 ×$\Delta f_{t}$ and 2 ×$\mu f_{t}$ respectively. Following are the observations that were noted from the first experiment set.

**Figure 5:**
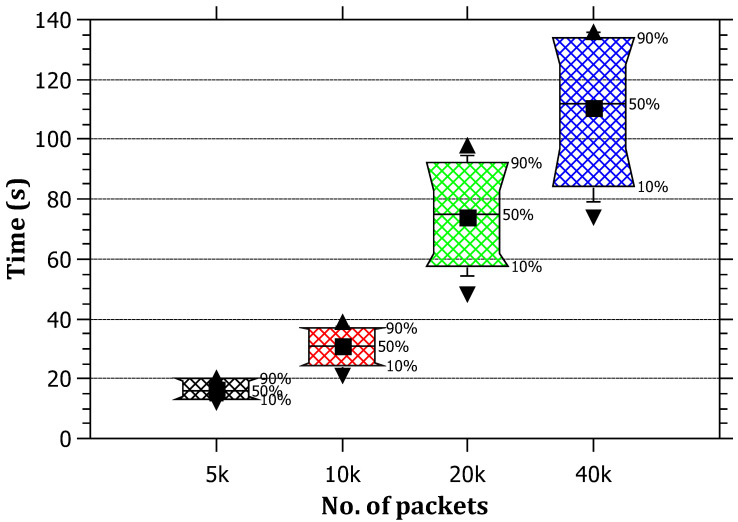
The relationship between the amount of traffic and the average detection time of the attack.

The results in Figure 5 show that the attack detection time is proportional to the amount of traffic. This is intuitive because, in order to achieve the condition that triggers the IP traceback algorithm at the edge router, a significant amount of malicious SYN packets needs to be injected. Therefore, the number of packets that must be forwarded before the detection of the attack will increase combined with the processing time at the edge routers. The tracing process requires more SYN packets destined to the victim before triggering the RAPT algorithm.

**Figure 6:**
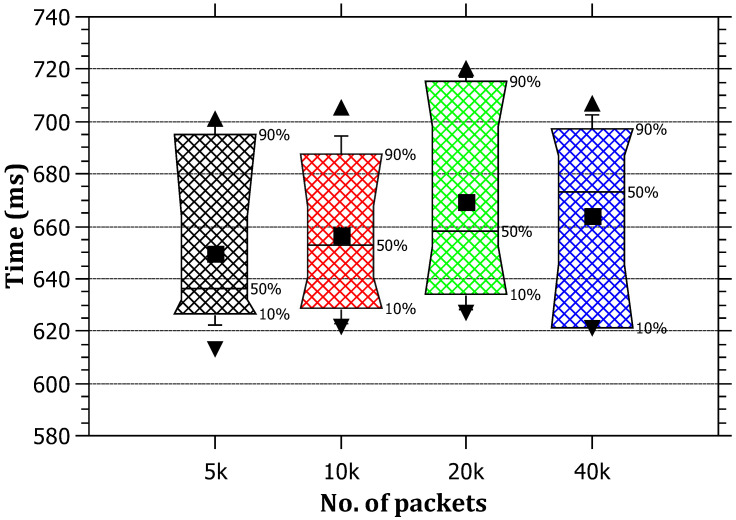
The relationship between the amount of traffic and the tracing time.

It was noted that the attack tracing time is not influenced by the amount of traffic generated at the edge routers. The reason behind this is that the tracing process needs one tracing packet per attack path; as a result, the tracing time is only for one packet (TracePack). The RAPT algorithm does not depend on the amount of traffic as much as it depends on the distance between the attacker and the victim. Figure 6 depicts that it remains within certain boundaries ranging between 630 and 670 milliseconds on average. The obtained results are feasible to quickly identify malicious activity by the victim and take an action to drop requests coming from the source IP.

**Figure 7:**
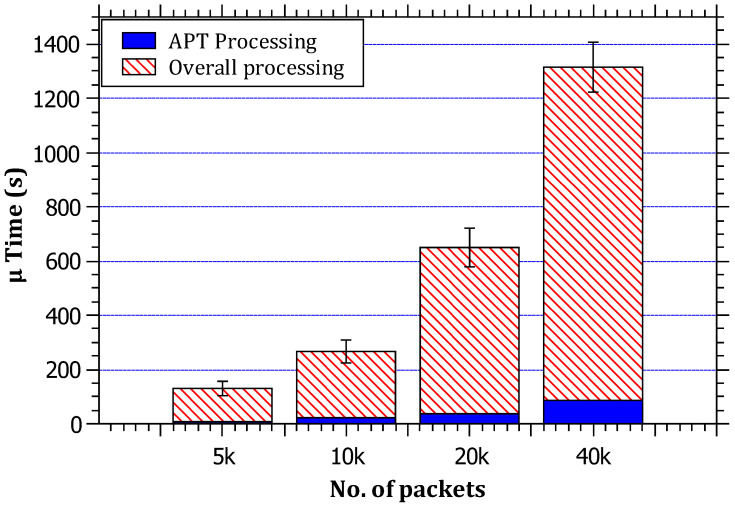
The relationship between the amount of traffic and the ratio of RAPT algorithm processing time to the total simulation processing time.

In Figure 7, it can be noticed that the ratio of the average processing time taken by the RAPT algorithm to the total simulation processing time does not exceed 9% in its most extreme cases, which are acceptable rates, although not optimal. The results also reveal that, with the increase in the traffic, the overall processing time increases in a linear correlation. The reasoning for this is that the simulation time = processing time + packet routing time. The reason for the linear increase in simulation time can be attributed to the packet routing time, and this is normal because every packet needs routing at every router on the path; therefore, it can be concluded that the processing burden does not constitute a large proportion of the path simulation time.

### 4.4. Second Experiment Set with Different Topologies

This experiment set examined the effect of changing the attack distance (hop count) on each of the tracing time, detection time, and processing time. Different topologies were considered with different distances between the attack source and the victim. The Waxman model proposed in [40] is used to create network topology. This Waxman model is a variant of the Erdös–Renyi random graph model in [41] with additional characteristics that are specific to the network. The amount of traffic generated was set to 3000 packets for each of the edge routers. The distances considered are 3, 6, 9, 12 and 15 hops. The rest of the parameters were set as in the first experiment set. The simulator was run 15 times for each of the distances, and the results were averaged.

**Figure 8:**
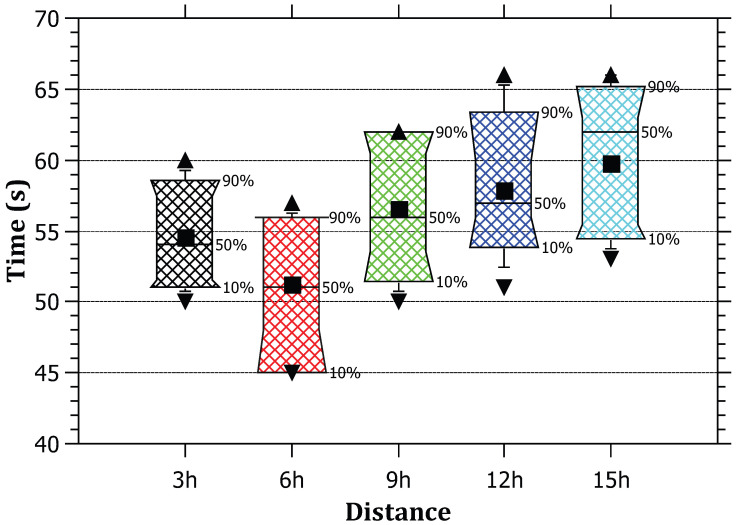
The relationship between the distance of the attack and its detection time.

The results in Figure 8 show that the relationship between the attack distance and the average detection time is almost independent of the distance and shows no increase in the detection time as the distance increases. This is due to the dependence on the edge routers to detect and initialize the RAPT algorithm and not the distance between the daemon and the victim. This is an advantage to the RAPT algorithm where, unlike other algorithms (as will be shown in the comparison with related work Section 4.5), the attack discovery is performed at the edge routers instead of the victim. This makes use of distributing the attack discovery burden on the network edge to make it proactive.

**Figure 9:**
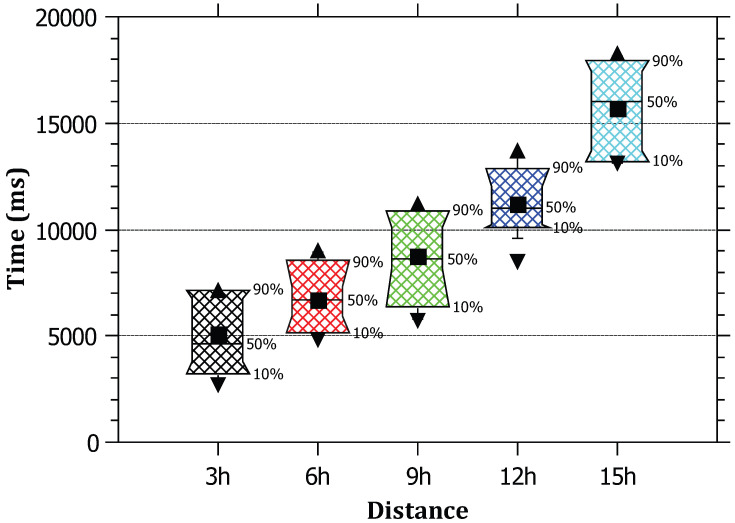
The relationship between the distance of the attack and the attack tracing time.

In Figure 9, the results indicate that the relationship is almost linear between the attack distance and the attack tracing time. This is due to the need of sending TracePack from router to router on the path. Recall that TracePack is reliably sent using hop-by-hop TCP connections to guarantee the arrival of TracePack. This adds extra delay for tracing. Moreover, packet loss of TracePack will add more delay. However, considering the average hop count over the Internet to be around 15 hops [42], and the tracing time is acceptable being around 15 s.

**Figure 10:**
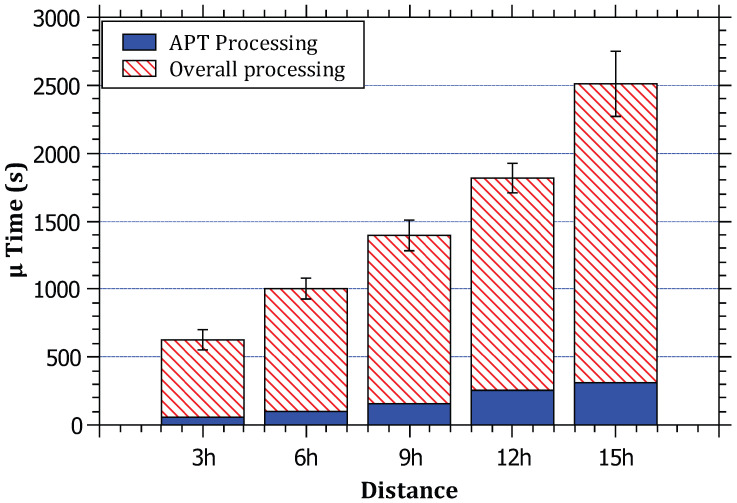
The relationship between the distance of the attack and the ratio of RAPT algorithm processing time to the total simulation processing time.

In Figure 10, it can be noted that the relationship between the attack distance and the processing time ratio to the total simulation time is almost logarithmic. This is because the increase in the attack distance corresponds to an increase in the processing time. Since the processing time is the sum of the processing time for each router (edge + core) on the path between the daemon and the victim, it can be inferred that this increase is reasonable due to the increase in the number of core routers on the path. Recall that, for each attack path, there is one edge router that demands more computations (See Algorithm 1) compared to negligible computations at the core routers (see Algorithm 2). The longer the distance is, the increase in the processing time slightly increases since the dominant amount of processing time is at the edge router that is executed only once per attack path.

### 4.5. Comparison with Related Work

In this set of experiments, the proposed RAPT algorithm was compared with TAPPM [21] scheme, which is a collaborative DDoS mitigation framework based on traffic awareness and IP traceback. The authors were motivated by the shortcoming when a DDoS occurs, and the upstream routers might drop most of the traffic before reaching the victim [43]. The authors designed TAPPM for IP traceback and traffic awareness. Using TAPPM, the victim can locate and discover hit routers. The victim then selects the hit routers that are on the attack path as collaborators to limit the forwarding rates aiming to achieve effective and rapid-response against DDoS. The main features of the TAPPM scheme are described below:

1. The scheme marks packets with tags carrying the traffic semantics and path information, so that the victim can perceive the distribution of the traffic routed to itself;
2. The DDoS traffic discovery is based on traffic volume changes, which allows the victim to trace the attack source generating the malicious traffic and locate the attack point;
3. The scheme collaboratively mitigates DDoS based on traffic awareness and IP traceback, which enables the victim location attack points and selecting some hit routers to act as collaborators.

**Figure 11:**
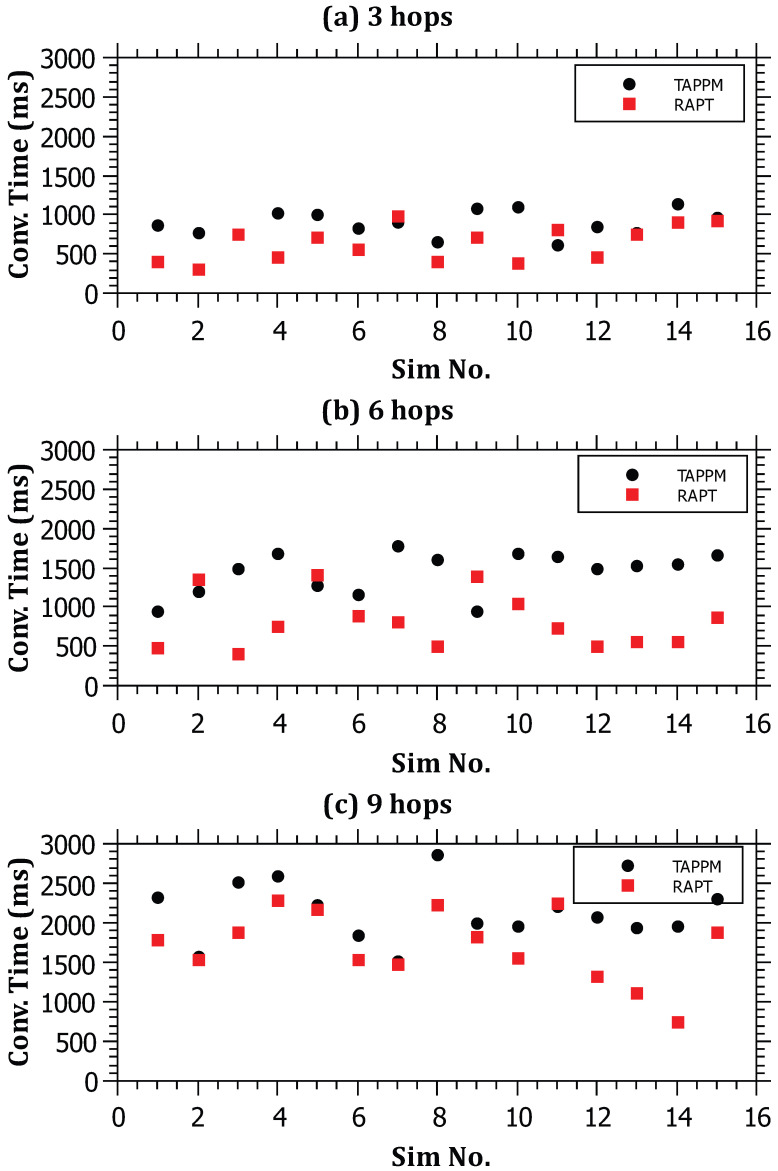
The relationship between the distance of the attack and the convergence time for (**a**) 3 hops; (**b**) 6 hops; and (**c**) 9 hops.

**Figure 12:**
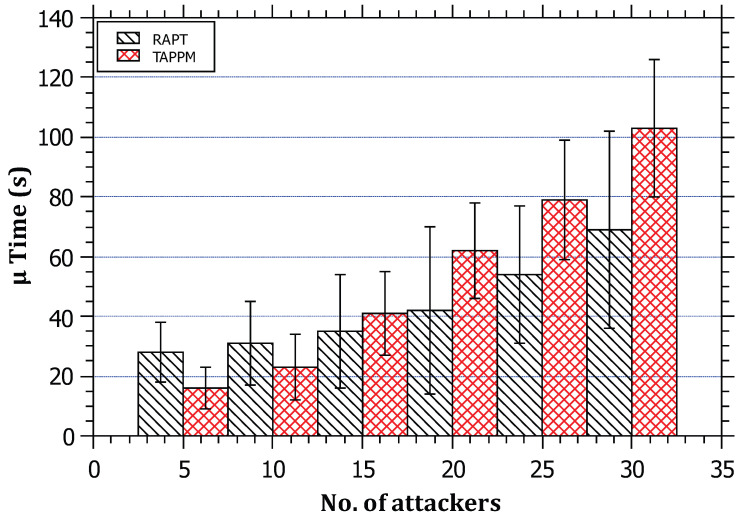
The relationship between the distance of the attack and its detection time.

For the sake of comparison, the following performance metrics were considered. The parameters’ values are similar to the first set of experiments unless explicitly mentioned in the results’ discussions:

1. **Convergence Time**: This is the time elapsed since the start of attack packets being generated from the attacker(s) until the building of the attack path. The performance of RAPT was compared with TAPPM by running the simulations 15 times over multiple distances, namely, 3, 6, and 9 hops. The results in Figure 11 show that the convergence time is proportional to the distance for both algorithms. This is predictable as the routers on the path need to perform computations and communications for both algorithms; however, RAPT outperforms TAPPM since the attack path will require only one packet to travel between the attacker and the victim. The TAPPM algorithm requires more time and packets from the attacker to realize the attack and select the hit routers, which will mark a significant amount of packets that will be received by the victim to rebuild the attack path.
2. **Robustness**: This metric is used to measure the resilience and scalability of the algorithm against large-scale DDoS attacks. The robustness of RAPT and TAPPM was evaluated in terms of scalability by increasing the number of attackers, that is, when the number of attackers exceeds 15 attackers. Both of the algorithms were evaluated by varying the number of attackers between 5 and 30 running the simulator 15 times for each algorithm and number of attackers. The amount of traffic generated from each attacker is fixed to 5000 packets. The obtained results as depicted in Figure 12 show that, when the number of attackers is less than 15, the average trace time per attack path is better for the TAPPM. This can be due to the fact that, when the number of attackers is low, the attack paths will definitely be low; therefore, less generated attack packets need to be injected in the attack path, which yields a small number of hit routers being collaborative. However, as the number of attackers increases, the average tracing time for the RAPT algorithm significantly performs better. The reason behind this is that the RAPT algorithm is resilient against multi-source/multi-path attacks, thanks to the use of a single tracing packet per path yielding parallel trace packets reaching the victim from multiple sources. Each path is tracked separately without relying on other paths, whereas in the TAPPM algorithm, when the number of attackers increases, the need for a larger number of packets to be marked will increase significantly causing the dramatic increase in average tracing time.
3. **Computation overhead at the victim**: An analytical comparison for both of RAPT and TAPPM is provided. Since the TAPPM algorithm relies on receiving significant amounts of marked packets at the victim where, in the case of large-scale DDoS, the edge-id fragments converge at the victim causing a bottleneck effect. To rebuild a single attack path, the victim has to perform computations on the hashed values embedded in the packets. These computations will cause a heavy burden to be put on the victim extracting different attack paths from multiple sources. Two factors are influencing the performance of TAPPM, the distance and the number of attackers/paths. These two factors negatively affect the performance as either or both increase in amount. The victim must try all combinations in the correct order, which makes it become like solving a puzzle. To elaborate more on this, in Figure 2, the edge-id of R1 and R6 would result in in eight fragments and eight more fragments for the edge-id of R2 and R6. Using combinations, the victim must arrange the correct fragments of each edge-id. Considering this scenario, but in a broader and more complex manner, by increasing the distance on the one hand and increasing the number of attackers on the other hand, this puts a heavy burden on the victim. The proposed RAPT algorithm will only require to recognize the trace packet, know the structure of it, and extract the attack path.
4. **Backwards Compatibility**: An analytical comparison between the RAPT and TAPPM algorithms is provided. Looking through the TAPPM algorithm, it was noted that it violates the IPv4 header specifications for every packet flagged by the hit routers on the path, when the distance between the attacker and the victim increases, the number of packets flagged by the hit routers increases, and this leads to a considerable amount of violations of IPv4 header specifications. However, in the proposed RAPT algorithm, the violation is only for one packet regardless of the distance between the victim and the attacker. Moreover, this packet can be a fragment where the RAPT algorithm uses the value stored in its identification field without the need to overwrite it. This advantage makes the RAPT algorithm not need to change the value of the identification field in the fragment; therefore, it does not lead to a problem in assembling the fragments. If the packet is not a fragment, RAPT changes the value of the identification field (which is set to zero) since it is unused because the packet is a single unit that is not fragmented.

## 5. Conclusions and Future Work

Since the spread of DoS/DDoS attacks in the early 2000s, they were and are still causing nuisance and financial losses to famous businesses. The Internet community both in academia and industry proposed a plethora of solutions. Heretofore, efficient and viable solutions are still on the research focus. In this work, the RAPT algorithm was proposed to efficiently solve the SYN-Flood DDoS attacks. The algorithm is deployed on edge and core routers enabling them to initiate the trace packet that accompanies the attack. The edge routers collect statistical information about the features and patterns of the traffic, which are used to predict if an attack is potentially taking place. The main characteristics of the RAPT algorithm are being lightweight and robust, having efficient detection and tracing time, and backward compatibility with IPv4, and it does not introduce extra traffic in the Internet. Extensive simulation runs were used to evaluate the efficiency of the RAPT algorithm. The obtained results prove the aforementioned characteristics; moreover, it outperforms the relevant TAPPM scheme. In the proposed RAPT algorithm, there are still some issues that need to be addressed in future work, such as the authentication of tracing packets where an attacker may inject forged tracing packets that might accuse legitimate hosts. Additionally, the solution requires modifications to the routers’ functionality of Autonomous Systems (AS). Inconsistency with IPsec [44] that authenticates the IP header must be considered. All of these mentioned issues can be motivating for future work that builds on the RAPT algorithm.

## Data Availability

Not applicable.
